# Child injured by suspected catfish (*Cetopsis* sp.) bite in river, Humaitá, Amazonas, Brazil

**DOI:** 10.1590/0037-8682-0458-2019

**Published:** 2020-04-22

**Authors:** William Lesimann, Tasso Queiroz, Luís Marcelo Aranha Camargo

**Affiliations:** 1 Centro Universitário São Lucas, Porto Velho, RO, Brasil.; 2 Centro de Pesquisa em Medicina Tropical, CEPEM/SESAU, Porto Velho, RO, Brasil.; 3 Universidade de São Paulo, Instituto de Ciências Biomédicas, Monte Negro, RO, Brasil.; 4 Conselho Nacional de Desenvolvimento Científico e Tecnológico, Instituto de Ciência e Tecnologia/CNPq EpiAmo, Porto Velho, RO, Brasil.

**Keywords:** Catfish, *Cetopsis* sp., Amazônia, Traumatic Injury

## Abstract

We present the first recent reported case of traumatic injury caused by catfish in the Americas. Although 66.2% of fish-related injuries occur in the Amazon Region, they are scarcely reported. We report a traumatic injury in a 2-year-old boy who entered Madeira River. The use of traditional methods to treat the injury and his incomplete vaccination history reflect the weakness of the health system. Further, the fact that it was the second time that such an incident had occurred in the locality in 3 weeks during the dry season suggests that this could be a frequent occurrence during this period.

## INTRODUCTION

Injuries caused by fish in the Amazon Region are relatively frequent but are rarely reported. Data from the Brazilian Ministry of Health on reporting cases of fish injuries in Brazil are recorded by SINAN (Notification of Injury Information System). Cases are most likely underreported either as a result of most victims not seeking hospital care or failure to fill in the notification forms and/or such events occurring in remote areas. Between 2007 and 2013, 4,118 injuries were registered in SINAN, of which 88.7% were caused by venomous species, particularly stingrays, and 11.3% were caused by trauma and/or poisoning from an unidentified species. Around 66.2% of cases occurred in the northern region of Brazil (Amazônia)^1^ ^-^ ^3^.

**FIGURE 1:**
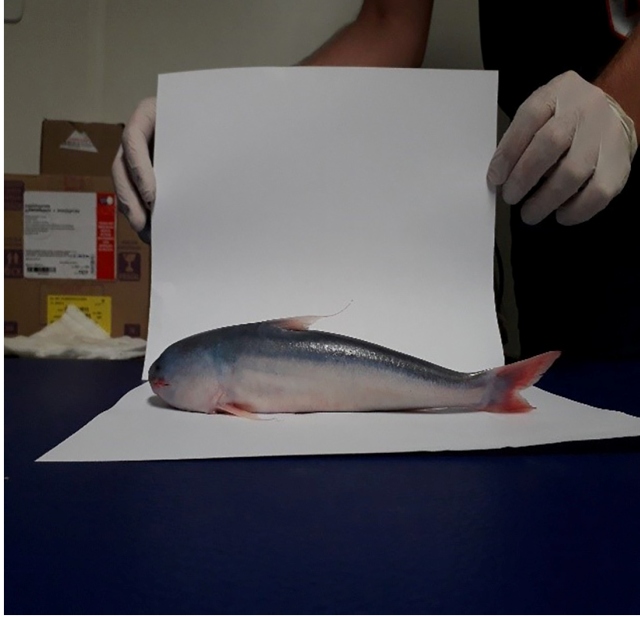
Lateral view of a 25 cm "Candiru-Açu" (whale catfish) . Caracará locality, Humaitá municipality, Amazonas State.

**FIGURE 2:**
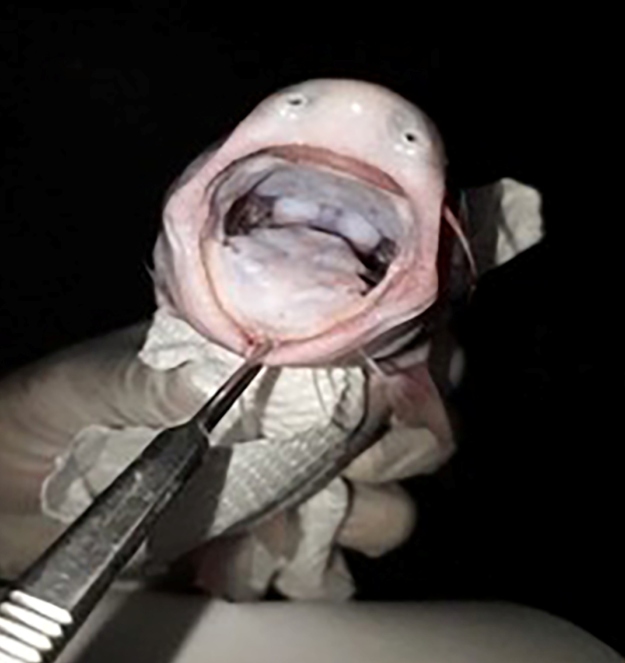
Mouth of "Candiru-Açu" (whale catfish). Caracará locality, Humaitá municipality, Amazonas State.

The species “Candiru-Açu” (Figure 1 and Figure 2), from the catfish group (Siluriformes Order), belongs to the Cetopsidae family. The “Candiru-Açu” species is often confused with the “Candiru” species from the Trichomycteridae family, which is known to feed on blood and penetrate the human urethra, as they are smaller than the species belonging to Ceptosidae. “Candiru-Açu”, which can measure up to 30 cm in length, is found throughout the Amazon basin where the rivers are laden with sediment. The species described is most likely *C. candiru* or *C. coecutiens,* which have been reported to be present in the Amazon Basin and in the upper Madeira region, including Humaitá municipality. This species also has a single row of incisional teeth, as observed by the research team. It often feeds on dead animals (and humans), penetrating the body and feeding on its internal organs^4^ ^-^ ^6^.

## CASE REPORT

**FIGURE 3:**
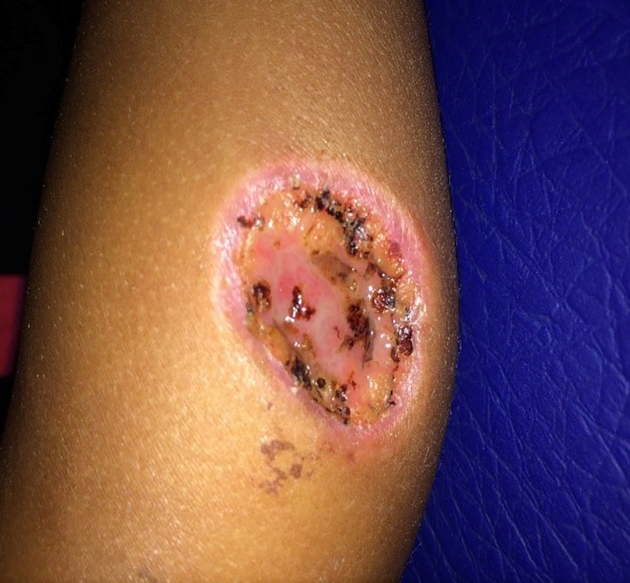
Injury by "Candiru-Açu" (whale catfish) bite, 3 × 3 cm^2^ of the calf. Caracará locality, Humaitá municipality, Amazonas State.

This study reports a suspected traumatic injury (Figure 3) caused by the “Candiru-Açu” (*Cetopsis* sp*.*) species in a 2-year-old child from a river community near the Madeira River in Caracará (W62º 30,159´; S 06º 44,780 S) Humaitá, Amazonas State, Brazil. The case was reported as a result of a visit by the health team from the Humaitá municipality and researchers from the University of São Paulo on October 16, 2019. The child, accompanied by the parent, sought out medical assistance 20 days after the injury. At the time, a consent form was signed for the case report and for photographic records. The patient’s mother stated that her son, JMSS, a 2-year-old boy who was born in and is a resident of the riverine locality, entered the Madeira River to bath along with her. Before reaching the floating wooden platform that is used to wash dishes and clothes and to bath on the riverbank, the child entered the water and soon afterward began to cry and shout. This prompted the mother to take her son out of the river, after which she saw a "candiru" on the child's leg, similar to the one pictured below. The “candiru” soon loosened its hold, leaving behind a deep circular lesion with slight hemorrhage on the left calf (3 × 3 cm^2^ in diameter) of the child. They then went home, where the mother made a saline solution for asepsis (one soup-spoon of salt in one cup of water) and used it to wash the lesion 3 times a day. They also used a home remedy, which involved rinsing the lesion with a watery solution made with guava bark, cashew bark, *Syzygium cumini* bark, and “dragon’s blood bark” as well as plant sap made from “dragons blood bark”. The latter was only applied once. The next day, the parent reported that purple and itchy blemishes had appeared in the palmar region of the left and right hands, while itchy papules had appeared simultaneously on the abdomen. Saltwater asepsis was performed for 2 days and the symptoms subsided. The mother reported that she used sulfadiazine as a topical treatment; she washed the wound with water, dried it, then crushed the sulfa tablet and then placed it on the lesion. The mother also reported that her son had fever on the 3^rd^ night (not measured). She said that no medication was used and that the fever had spontaneously resolved by morning. She also denied the presence of other associated symptoms (diarrhea, headache, and vomiting). The child showed no signs of comorbidity and a physical examination showed no changes, except for the lesion. The child had an incomplete vaccination history for hepatitis B, measles, and the tetravalent vaccine. The lesion was open with no phlogistic signs and no purulent discharge. There was granulation tissue around the border and at the bottom of the lesion. The health team decided to instruct the mother to wash the lesion 3 times a day with soap and water, and prescribed neomycin and bacitracin ointment to allow healing to occur by secondary intention. The child’s vaccinations were updated. The mother also reported that another person from the community (an adult male) had suffered a similar injury to his face while diving in the Madeira River during mining activities 3 weeks before.

## DISCUSSION

The manuscript presents the first reported case of a traumatic injury caused by catfish in the Americas in recent times. The injury is thought to have been caused by a fish from the Cetopsis family, as identified by the mother and the research team. Injuries by venomous or non-venomous fish in Amazônia are frequent and underreported. 

The rash on the hands and abdomen of the child likely occurred as a secondary effect of topical use of herbal plants and/or sulfadiazine; i.e., they are likely to be caused by an allergic reaction. There is little evidence to suggest that such a manifestation would come from any substance in the oral cavity of the fish. 

The use of local traditions for treatment of the injury and the delayed vaccination status of the child reflect the weakness of the local health system. The fact that it was the second time that such an incident had occurred during the dry season in 3 weeks in a locality with only 44 residents suggests that injuries caused by catfish might be quite a frequent occurrence during this period. Therefore, people should be warned about such risks while swimming/bathing in the Madeira River, especially during the dry season.
